# A Single Animal Species-Based Prediction of Human Clearance and First-in-Human Dose of Monoclonal Antibodies: Beyond Monkey

**DOI:** 10.3390/antib10030035

**Published:** 2021-09-05

**Authors:** Iftekhar Mahmood

**Affiliations:** Mahmood Clinical Pharmacology Consultancy, LLC., Rockville, MD 20850, USA; Iftekharmahmood@aol.com; Tel.: +1-301-838-4555

**Keywords:** allometry, antibodies, clearance, first-in-human dose, human-equivalent dose

## Abstract

These days, there is a lot of emphasis on the prediction of human clearance (CL) from a single species for monoclonal antibodies (mabs). Many studies indicate that monkey is the most suitable species for the prediction of human clearance for mabs. However, it is not well established if rodents (mouse or rat) can also be used to predict human CL for mabs. The objectives of this study were to predict and compare human CL as well as first-in-human dose of mabs from mouse or rat, ormonkey. Four methods were used for the prediction of human CL of mabs. These methods were: use of four allometric exponents (0.75, 0.80, 0.85, and 0.90), a minimal physiologically based pharmacokinetics method (mPBPK), lymph flow rate, and liver blood flow rate. Based on the predicted CL, first-in-human dose of mabs was projected using either exponent 1.0 (linear scaling) or exponent 0.85, and human-equivalent dose (HED) from each of these species. The results of the study indicated that rat or mouse could provide a reasonably accurate prediction of human CL as well as first-in-human dose of mabs. When exponent 0.85 was used for CL prediction, there were 78%, 95%, and 92% observations within a 2-fold prediction error for mouse, rat, and monkey, respectively. Predicted human dose fell within the observed human dose range (administered to humans) for 10 out of 13 mabs for mouse, 11 out of 12 mabs for rat, and 12 out of 15 mabs for monkey. Overall, the clearance and first-in-human dose of mabs were predicted reasonably well by all three species (a single species). On average, monkey may be the best species for the prediction of human clearance and human dose but mouse or rat especially; rat can be a very useful species for conducting the aforementioned studies.

## 1. Introduction

In recent years, the prediction of pharmacokinetic (PK) parameters from a single animal species such as rat, dog, or monkey to humans has been gaining momentum for both small and macro-molecules. Since clearance is an important PK parameter, most studies have focused on predicting clearance of drugs from animals to humans. These studies show that the monkey is the most suitable animal species to predict drug clearances in humans.

Like small molecules, there is a lot of emphasis on the prediction of human PK parameters, mainly clearance for monoclonal antibodies (mabs) from a single species [1,2,3]. These methods use a fixed exponent on body weight to predict CL of mabs in humans from monkey. Lin et al. [2] concluded that exponents 0.85 and 0.9 are needed to predict human clearance for mabs in humans from monkey for soluble antigens as well as membrane-bound antigens, respectively. Deng et al. [1] also found that, using monkey clearance and exponent 0.85, one can predict antibody clearance in humans. Oitate et al. [3] predicted human CL of mabs from monkey data. Clearance values of 24 mAbs (soluble or membrane-bound antigens) were analyzed. The authors used training data sets to determine the exponents of allometry for soluble mabs (*n* = 5) and membrane-bound mabs (*n* = 7). The mean exponent for soluble mabs was 0.79 (range = 0.62–0.98) and for membrane-bound mabs was 0.96 (range = 0.67–1.13). The authors then used 5 soluble mabs and 7 membrane-bound mabs to validate the predictive performance of their mean exponents obtained from the monkeys for the prediction of human CL. In the validation data set, the mean predicted-to-observed ratio for soluble mabs was 0.76 (range = 0.64–1.03) and for membrane-bound mabs was 1.15 (range = 0.78–1.54). The authors concluded that human CL of mabs can be predicted with reasonable accuracy (within 2-fold of the observed values) from monkey alone.

The works of Deng, Ling, and Oitate do indicate that using a fixed exponent (although different from one study to the other) and monkey CL, one can predict human clearance of mabs within a twofold prediction error. However, these studies only focused on evaluating monkey as the most suitable species, ignoring two other widely used species, namely, mouse and rat, for the PK studies of mabs. Therefore, the objectives of this study were as follows:

- to evaluate the suitability of mouse or rat for the prediction of human CL of mabs and compare with the predicted values from monkey;
- to predict the first-in-human dose of mabs based on a single species and compare it with the doses given to humans (dose range).

## 2. Methods

### 2.1. Prediction of Human Clearance

From the literature, the clearance values for mabs for mouse, rat, monkey, and humans following intravenous administration were obtained [1,4,5,6,7,8,9,10,11,12,13,14,15,16,17,18,19,20,21,22,23,24,25,26,27,28,29,30,31,32,33,34]. The following methods were used to predict clearance in humans from a single species. Since non-linearity across several doses of mAbs is a common observation, in this analysis, if there were different doses then the clearance of the dose was used which was in the linear range across the doses.

#### 2.1.1. Allometric Exponent-Based Method

The following allometric methods were used to predict clearance in humans from a single species using different allometric exponents (Equation (1)).

Predicted CL in humans = CL of the species × (70/weight of the species)^b^ (1)

where CL of the species is the clearance of mouse, rat, or monkey and 70 is the human body weight in kilograms (kg). ‘b’ is the allometric exponent and four exponents (0.75, 0.80, 0.85, and 0.90) were used in this analysis. Body weights for mouse, rat, and monkey used in this study were 0.02, 0.25, and 3.5 kg, respectively.

#### 2.1.2. Minimal PBPK Method (mPBPK)

In this method, five physiological parameters were used. These physiological parameters were liver and kidney weights, liver and kidney blood flow, and lymph flow rate (Supplementary Materials Table S1). Human physiological parameters were adjusted from mouse, rat, and monkey, and physiological factors were obtained for each species, as described in the Supplementary Materials (Table S2). Human clearance was predicted by Equation (2).

Predicted CL in humans = CL of the species × physiological factor of the species(2)

The physiological factor for mouse, rat, and monkey was 936, 130, and 10, respectively (Supplementary Table S2).

#### 2.1.3. Lymph Flow Rate

In this method, only lymph flow rate was used to predict human clearance. A physiological factor was obtained for each species, as described in the Supplementary Materials. Human clearance was predicted by Equation (3).

Predicted CL in humans = CL of the species × physiological factor of the species(3)

The physiological factor for mouse, rat, and monkey was 1009, 94, and 10, respectively (Supplementary Table S2).

#### 2.1.4. Liver Blood Flow Rate

A method to predict human clearance of small molecules following intravenous administration using liver blood flow was proposed by Ward et al. [35]. In this method, it was found that the monkey was the best species as compared with rat and dog to predict human clearance. A physiological factor was obtained as follows, Equation (4):

Physiological factor = human liver blood flow/animal blood flow(4)

In this study, the liver blood flow in mouse, rat, monkey, and human was 1.8, 13.8, 158 (3.5 kg monkey), and 1600 mL/min, respectively [36]. Based on Equation (4), a physiological factor in mouse, rat, and monkey was 889, 116, and 10, respectively, Equation (5) (Supplementary Table S2).

Predicted CL in humans = CL of the species × physiological factor of the species(5)

## 3. Prediction of Human Dose

### 3.1. First-in-Human Dose Estimation from Predicted Human Clearance

In a study, Mahmood et al. [37] used the predicted human clearance from animals (at least a three-species scaling) of small-molecule drugs for the selection of the first-in-human dose. The authors used several approaches for the estimation of first-in-human dose for small molecules. Later, Mahmood took the similar approach to predict clearance and the first-in-human dose for macromolecules [38,39]. Mahmood’s methods were based on the scaling (clearance) of at least three animal species. Since, in the current study, clearance was predicted from a single species, the predicted first-in-human dose was also based on a single-species scaling.

In the preclinical stage, animals were given several doses ranging from 0.1 to 20 mg/kg. Most of the species received a dose of 10 mg/kg or near 10 mg/kg. Since preclinical dose will have an impact on the first-in-human dose selection, in order to predict first-in-human dose, an animal dose of 10 mg/kg was used. If a 10 mg/kg dose was not available in animal species, then a dose nearest to 10 mg/kg (lower than 10 mg/kg) was used. However, there was one exception (EGF/r3), where a 16 mg/kg dose was given to the mouse and this was the only dose used in this analysis which was >10 mg/kg. Rat received 8 mg/kg EGF/r3 and there were no monkey data. In real life, one can predict human dose from all the doses (within the linear range) given to the animals and then use scientific judgment and experience to decide on the first-in-human dose. The following methods were used for the prediction of first-in-human dose.

#### 3.1.1. Method I: Linear Method

The following Equation (6) was used to estimate first-in-human dose by linear method.

Predicted first-in-human dose = Dose in animal × (human CL/animal CL)(6)

where dose and CL were in absolute numbers (not normalized to body weight). Human CL was predicted CL from a given species.

#### 3.1.2. Method II: Exponential Method

The following Equation (7) was used to estimate first-in-human dose by exponential method.

Predicted first-in-human dose = Dose in animal × (human CL/animal CL)^0.85^ (7)

where dose and CL are in absolute numbers (not normalized to body weight). The exponent 0.85 was used since this exponent was the best exponent for the prediction of human clearance (1).

#### 3.1.3. Method III: Human-Equivalent Dose (HED) Based on Body Weight 

The HED for a given species was estimated as follows (body weight-based), Equation (8):

HED (Dose/kg) = Animal dose/kg × (Animal weight in kg/human weight in kg)^0.33^ (8)

#### 3.1.4. Method IV: Human-Equivalent Dose (HED) Based on Predicted Human Clearance

This was a modified version of Equation (8).

The HED in for a given species was estimated as follows using exponent 0.33 and CL, Equation (9):

HED (Dose/kg) = Animal dose/kg × (Animal CL/predicted human CL)^0.33^ (9)

#### 3.1.5. Method V: Human-Equivalent Dose (HED) Based on Predicted Human Clearance

HED in for a given species was estimated as follows using exponent 0.25 and CL, Equation (10):

HED (Dose/kg) = Animal dose/kg × (Animal CL/predicted human CL)^0.25^ (10)

The predicted human clearance and first-in-human dose were compared with the observed human clearance and the doses given to humans, respectively.

## 4. Statistical Analysis

Predicted-to-observed CL ratio was calculated as follows, Equation (11):

Ratio = Predicted CL/Observed CL(11)

Number of mabs and percent of total number of mabs in a species for fold errors within 0.5–2, 0.5–1.5, >2 and <0.5 were calculated. In the literature, the predicted ratio between 0.5–2 is considered acceptable.

Average fold error (AFE), which is the log-transformed ratio of the predicted and observed clearance values, was also reported for each species. For AFE, a value of 1.0 indicates no prediction error, and AFE was calculated as follows, Equation (12):

AFE = 10^1/N∑log(CL_predicted_/CL_observed_)^ (12)

where AFE is average fold error, N is the number of observations, and CL_predicted_ and CL_observed_ are the predicted and observed clearance values, respectively.

## 5. Results

### 5.1. Prediction of Clearance of Mabs from One Species to Humans

There were 23, 21, and 25 mabs for mouse, rat, and monkey, respectively. In Table 1, the names of the mabs used in different species are provided. The results of the study are summarized in Table 2. In Table 2, the number of mabs and percent of total number of mabs in all three species for different fold errors and AFE are shown. The accuracy of the prediction of mab clearance varied from exponent to exponent and from species to species.

### 5.2. Mouse

In mouse, exponent 0.85 provided the best prediction of human CL (Table 2). Using exponent 0.85 and mouse CL of mabs, the predicted human CL of mabs was within a 2-fold prediction error for 18 (78%) out of 23 mabs. The AFE from exponent 0.85 was 0.82, indicating a mean under-prediction by 18%. There was one mab with a prediction error >2-fold and 4 mabs were with a prediction error <0.5-fold. The exponent 0.90 over-predicted (>2-fold prediction error) human CL of mabs in 8 (35%) out of 23 mabs. The AFE from exponent 0.9 was 1.23. The theoretical exponent 0.75 under-predicted (<0.5-fold prediction error) human mab CL in 15 (65%) out of 23 mabs. The AFE from exponent 0.75 was 0.36 (Table 2), under-prediction by 64%. In a practical world, both exponents 0.85 and 0.90 are acceptable because both over- and under-prediction are not very high.

Minimum PBPK (mPBPK), lymph flow rate, and liver blood flow provided similar results as exponent 0.85 (Table 2). The predicted human CL of mabs was within a 2-fold prediction error for 17 (74%), 17 (74%), and 16 (70%) for mPBPK, lymph flow rate, and liver blood flow, respectively. The AFE by mPBPK, lymph flow rate, and liver blood flow was 0.74, 0.80, and 0.71, respectively (Table 2). It is worth noting that 5 physiological parameters were used in mPBPK, but the overall result was not better when only a single physiological parameter such as lymph flow rate or liver blood flow was used for the prediction of human CL.

Overall, the analysis indicated that, exponent 0.85, mPBPK, lymph flow rate, or liver blood flow are reasonable methods to predict human clearance from mouse clearance.

### 5.3. Rat

In rat, like mouse, exponent 0.85 also provided the best prediction of human CL (Table 2). Using exponent 0.85 and rat CL of mabs, the predicted human CL of mabs was within a 2-fold prediction error for 20 (95%) out of 21 mabs. The AFE from exponent 0.85 was 1.1. The CL of only one mab was predicted with a >2-fold prediction error (no mab with prediction error of <0.5-fold). The exponent 0.90 over-predicted (>2-fold prediction error) human CL of mabs in 2 out of 21 mabs. The AFE from exponent 0.90 was 1.46. The theoretical exponent 0.75 under-predicted (<0.5-fold prediction error) human mab CL in 7 out of 23 mabs. The AFE from exponent 0.75 was 0.63 (Table 2).

Minimum PBPK (mPBPK), lymph flow rate, and liver blood flow provided similar results as exponent 0.85 (Table 2). The predicted human CL of mabs was within a 2-fold prediction error for 19 (90%), 19 (90%), and 20 (95%) for mPBPK, lymph flow rate, and liver blood flow, respectively. The AFE by mPBPK, lymph flow rate, and liver blood flow was 1.19, 0.86, and 1.06, respectively (Table 2).

### 5.4. Monkey

All four exponents provided good prediction (within a 2-fold prediction error) of human clearance. Using exponents 0.90, 0.85, 0.80, and 0.75 and monkey CL of mabs, the predicted human CL of mabs was within a 2-fold prediction error for 23 (92%) out of 25 mabs. The AFE for exponents 0.90, 0.85, 0.80, and 0.75 was 1.03, 0.89, 0.76, and 0.66, respectively. The CL of only two mabs in humans were predicted with a <0.5-fold prediction error and there was no mab whose predicted human clearance exceeded 2-fold (Table 2). Based on AFE and a 2-fold prediction error, exponent 0.90 produced the best results in the monkey (Table 2). However, for all practical purposes, all four exponents provided good prediction (within a 2-fold prediction error) of human clearance from monkey. Based on the AFE, exponent 0.90 or 0.85 should be used for the prediction of human clearance of mabs from monkey.

Minimum PBPK (mPBPK), lymph flow rate, and liver blood flow provided similar results as exponent 0.85 or 0.90 (Table 2). The predicted human CL of mabs was within a 2-fold prediction error for 23 (92%), 23 (92%), and 23 (92%) for mPBPK, lymph flow rate, and liver blood flow, respectively. The AFE by mPBPK, lymph flow rate, and liver blood flow was 0.70 (under-prediction by 30%).

Overall, the results of the study indicate that, on the whole, exponent 0.85 is the most suitable exponent to predict human mab CL from mouse and rat (Table 2). All 4 exponents produced the similar prediction error for monkey. Based on the AFE, the theoretical exponent 0.75 under-predicted the human CL of mabs in all three species (approximately 34% to 64%). Based on AFE, exponent 0.90 over-predicted the human CL of mabs in mouse (23%) and rat (46%), but not in monkey. All seven methods indicated that the predicted human clearance is comparable between rat and monkey. Exponent 0.85 from the perspective of 2-fold error and AFE appears to be the best method among all 7 methods evaluated for all three species. Of the two rodents (mouse and rat), the predicted human clearance was better by the rat (95%) than the mouse (78%). The predicted human clearance of mabs by monkey or rat was comparable.

### 5.5. Prediction of First-in-Human Dose from One Species

There were 13, 12, and 15 mabs for which first-in-human dose was predicted from mouse, rat, and monkey, respectively. Predicted and observed human dose by different methods are summarized in Table 3. The results of the study indicated that the first-in-human dose was predicted with reasonable accuracy by all 3 species (Table 3). The main criterion for successful human dose prediction was whether or not the predicted human dose fell within the observed doses given to humans. For most of the mabs, the doses (within species) given to animals and humans widely varied. The five approaches predicted different doses in humans. A general characteristic of predicted human dose was that the highest dose was predicted in humans using predicted human clearance and exponent 1.0 (method 1 or linear method) from all three species (Table 3). The lowest human dose was predicted by HED using body weight and exponent 0.33. This was a common observation across all three species (Table 3). It was also noted that the predicted human dose by mouse was the lowest and the highest by the monkey.

The advantage of these five methods is that one can obtain a wide range of doses and can use scientific judgment, experience, and knowledge to decide about the first-in-human dose selection. It should be noted that the predicted doses by these methods were at the lower end of the doses which were administered to humans. This is ideal because the objective is to select first-in-human dose, which is safe, and then escalate the dose accordingly.

Human dose prediction is also dependent on the dose given to animals. For example, cetuximab (Table 3) dose was substantially under-predicted from both mouse and monkey (rat data were not available). The reason for this was that very low doses were given to mouse (0.04, 0.25, 1 mg/kg) and monkey (0.026, 0.08, 0.26 mg/kg).

## 6. Discussion

This analysis indicates that, for a single-species scaling, mouse or rat can be as useful as monkey. Of the two rodents (mouse or rat), rat appears to be more accurate in its predictive performance of clearance than mouse and as accurate as monkey (Table 2). For the prediction of human dose, however, mouse was as good as rat or monkey.

The caveat of a single exponent and a single-species scaling for the prediction of human CL of mabs should be recognized. A single exponent is not necessarily the most optimal exponent for a single-species scaling and carries high uncertainty [37,38,39]. For example, for pertuzumab and bevacizumab, the prediction error in clearance from mouse and exponent 0.9 was 6% and 107%, respectively (38% from exponent 0.85 for both mabs). From rat (exponent 0.9), the prediction error in clearance for pertuzumab and bevacizumab was 54% and 17%, respectively. From monkey (exponent 0.9), the prediction error in clearance for pertuzumab and bevacizumab was 15% and 19%, respectively.

Even in monkey, when different exponents were used, a wide range of prediction errors were noted for most of the mabs. For example, for anti-CD40, the percent prediction error ranged from 5% to 65% across different exponents. A similar phenomenon was noted with mouse and rat. Although, on average, it appears that monkey is the best species among mouse, rat, and monkey for the prediction of human CL for mabs, it is not necessary that for a given individual mab monkey will always perform better than the other two species. For example, for anti-CD40, the prediction error (exponent 0.85) from mouse, rat, and monkey was 37%, 13% and 42% percent, respectively. Similarly, for RSHZ19, the prediction error (exponent 0.85) from rat and monkey was 8%, and 31% percent, respectively. On the other hand, it also appears that in most cases the prediction of human CL of mabs may be more accurate from monkey than mouse or rat. Since a 2-fold prediction error is widely accepted, both for mouse and rat especially, rat is a comparable species with monkey for the prediction of human CL of mabs.

Considering that there is a systematic pattern in the predicted mab clearance in humans from mouse and rat by the allometric exponents, one can use these exponents for potential advantage. The exponent 0.75 and exponent 0.90 produced the lowest and the highest predicted CL values of mabs, respectively, from all three species. One can use all 4 exponents as shown in this study and this will give a range of CL values (or mean clearance values), which then can be tailored using experience and scientific judgment for the first-in-human dose selection. Especially, this approach may be useful from mouse data.

In this study, normal mouse data were used, but these days the focus is also on using transgenic mouse. The importance of transgenic mice in preclinical and clinical studies has been highlighted in many studies [40,41]. In a study, Valente et al. [40] emphasized the use of humanized FcRn transgenic mice to predict the PK of mAbs in humans. In their study, the authors used humanized FcRn transgenic mouse (homozygous Tg32 and Tg276) and non-human primate (NHP) models and showed that the Tg32 mouse model can replace NHP models. The allometric exponent for clearance scaling from Tg32 mice to NHPs was estimated to be 0.91 for all antibodies.

Binding of immunoglobulins (IgGs) with the neonatal Fc receptor (FcRn) protects them from degradation, resulting in higher concentrations of IgGs. This increases the half-life and decreases the clearance of IgGs. It should be noted, however, that there are interspecies differences in the binding of IgGs with FcRn. Human IgG1 binds cynomolgus monkey FcRn with a 2-fold higher affinity than human FcRn, and binds both mouse and rat FcRn with a 10-fold higher affinity than human FcRn [42].

Estimation of a first-in-human starting dose for clinical trials of new drugs (small or large molecules) in healthy volunteers or patients is very important, since a low starting dose will prolong dose optimization, and a high starting dose may cause serious toxicity [43]. However, despite the importance of this task, there is no consensus regarding the best approach for estimating the first-in-human dose [44,45]. One of the methods is to convert an animal no observed adverse effect level (NOAEL) to the human-equivalent dose using appropriate scaling factors, followed by application of a safety factor [46]. However, determination of the appropriate animal NOAEL is a difficult and time-consuming task, depending on several factors such as duration of treatment, dose selection, and species. The choice of an appropriate scaling factor also involves considerable uncertainty. In order to select the first-in-human dose, Reigner and Blesch [44] suggested the use of the lowest AUC at the NOAEL when a drug is given to several species as well as the predicted human clearance (dose in humans = AUC × predicted human clearance). Thus, the proposed approach of Reigner and Blesch for first-in-human dose selection takes into account both the animal toxicity based on NOAEL and PK parameter, namely, clearance.

The antibody disposition or clearance is generally described as ‘endogenous’ or non-specific or target-mediated. Non-specific clearance of antibodies is due to the degradation by pinocytosis following cellular endosomal uptake and subsequent lysosomal proteolytic degradation into amino acids or smaller peptides [42]. In the endosome, antibodies can be protected from degradation by binding to the neonatal Fc receptor (FcRn). It was noted that, in FcRn-knockout (FcRn KO) mice, antibody clearance was >8 times higher than in wild-type mice [47]. Besides these two mechanisms of degradation of antibodies, studies have been conducted to demonstrate that antibodies can bind with the tissues and can be degraded in the tissues [47]. Through a physiologically based PK modeling, Eigenmann et al. [47] attempted to quantify tissue-specific intrinsic clearances of monoclonal antibody. The authors found that the major tissues for antibody catabolism in mice were liver (30 and 41%), skin (25 and 27%), and muscle (19 and 10%), for FcRn wild type and FcRn^−^ respectively. These tissues alone represented 74% and 78% of the total clearance of the antibody. The formation of anti-drug antibody and off target binding can also impact the PK of antibodies. These factors may be important and should be considered for the design of first-in-human dose selection.

For the first-in-human dose selection, the single-species method produced reasonably accurate prediction by using predicted human clearance from all three species. An accurate prediction in this case means the predicted dose was within the given human dose range. In almost all cases, the predicted first-in-human dose was towards the lower side, which is more desirable. From Table 3, it appears that using one species and a fixed exponent (0.33), the predicted human dose using human body weight (HED) is more conservative than the dose predicted using predicted human CL. However, the decision for the selection of first-in-human dose may depend on experience and scientific judgment. In the current analysis, where available, a maximum dose of 10 mg/kg given to animals was used. However, an investigator may want to use a much lower dose given to animals and then proceed from there. However, a very low starting dose in humans may not be of any practical value if one has to increase the human dose to a therapeutic level through many steps. It should also be noted that the pharmacokinetic studies of mabs are generally conducted in healthy animals and the first-in-human dose may not be always in healthy subjects.

## 7. Conclusions

Due to wide belief that monkey is the best species to predict human clearance and first-in-human dose, the investigations in the rodents were not undertaken or ignored. The current study indicates that mouse or rat, especially rat, can replace monkey for achieving the aforementioned goals. Mouse and rat are cheaper than monkey and also much easier to handle for preclinical studies than monkey.

Overall, the clearance and first-in-human dose of mabs were predicted reasonably well by all three species. On average, monkey may be numerically the best species for the prediction of human clearance and first-in-human dose selection, but both mouse and rat, especially rat, can be a very useful species for conducting the aforementioned studies. The use of mouse or rat in first-in-human dose selection is practical and cost- and time-effective without compromising the accuracy.

## Figures and Tables

**Table 1:** Name of the mabs used in the analysis.

Mabs	Type	Target	Mouse	Rat	Monkey	References
Pertuzumab	Human IgG1	HER-2	Yes	Yes	Yes	[1]
Bevacizumab	Human IgG1	VEGF	Yes	Yes	Yes	[1]
Trastazumab	Human IgG1	HER2	Yes	NA	Yes	[1]
Omalizumab	Human IgG1	IgE	Yes	Yes	Yes	[1]
GNE mAB S	NA	NA	Yes	Yes	Yes	[1]
GNE mAB T	NA	NA	NA	Yes	Yes	[1]
GNE mAB X	NA	NA	Yes	Yes	Yes	[1]
GNE mAB Y	NA	NA	Yes	Yes	Yes	[1]
GNE mAB Z	NA	NA	Yes	Yes	Yes	[1]
GNE mAB V	NA	NA	Yes	NA	Yes	[1]
Dacetuzumab	Human IgG1	CD40	Yes	Yes	Yes	[1]
RSHZ19	Human IgG1	RSV	Yes	Yes	Yes	[6,7]
Lenercept	Human IgG1	TNF	NA	Yes	Yes	[8]
Cetuximab	Chimeric IgG1	EGF	Yes	NA	Yes	[9,10,11]
CTLA-4Ig	Human IgG1	TNFα	Yes	Yes	Yes	[12,13,14,15]
CD4-IgG	Human IgG1	CD4	NA	Yes	Yes	[16,17]
MNRP1685A	Human IgG1	neuropilin-1	Yes	Yes	Yes	[18]
Canakinumab	Human IgG1	IL-1β	Yes	NA	Yes	[19]
Onartuzumab	Human IgG1	MET	Yes	NA	Yes	[20,21]
EGF/r3	IgG2a	EGF	Yes	Yes	NA	[22]
CNT05825	human anti-interleukin-13	IL-13	NA	Yes	Yes	[23]
Pembrolizumab	Human IgG4	PD-1	Yes	NA	Yes	[24,25,26]
Infliximab	Chimeric IgG1	TNFα	Yes	Yes	NA	[27,28]
Avelumab	Human IgG1	PD-1	Yes	NA	Yes	[29]
Adalimumab	Human IgG1	TNFα	Yes	Yes	Yes	[30,31,32]
Dupilumab	Human IgG4	IL-4	NA	Yes	Yes	[33]
Erlizumab	Human IgG1	VEGF	Yes	Yes	Yes	[34]
Rituximab	Chimeric IgG1	CD20	Yes	Yes	NA	[34]

NA = not available because the marketed or original names of the mAbs is not known. HER2 = human epidermal growth factor receptor 2, VEGF = vascular endothelial growth factor, RSV = respiratory syncytial virus, TNF = tumor necrosis factor, EGF = epidermal growth factor, CD4 = cluster of differentiation 4, IL = interleukin, PD-1programmed death-1.

**Table 2:** Prediction of human clearance from preclinical species by different methods.

Fold Error	Exponents				mPBPK	Lymph Flow	LBF
	0.90	0.85	0.80	0.75			
**Mouse (*n* = 23)**							
0.5–2 fold	14 (61%)	18 (78%)	11 (48%)	8 (35%)	17(74%)	17 (74%)	16 (70%)
0.5–1.5 fold	12 (52%)	15 (65%)	11 (48%)	8 (35%)	15 (65%)	14 (61%)	14 (61%)
>2 fold	8 (35%)	1 (4%)	0 (0%)	0 (0%)	0 (0%)	1 (4%)	0 (0%)
<0.5 fold	1 (4%)	4 (17%)	12 (52%)	15 (65%)	6 (26%)	5 (22%)	7 (30%)
AFE	1.23	0.82	0.54	0.36	0.74	0.80	0.71
**Rat (*n* = 21)**							
0.5–2 fold	19 (90%)	20 (95%)	18 (86%)	13 (62%)	19 (90%)	19 (90%)	20 (95%)
0.5–1.5 fold	13 (62%)	18 (86%)	12 (57%)	12 (57%)	17 (81%)	18 (86%)	19 (90%)
>2 fold	2 (10%)	1 (5%)	1 (5%)	1 (5%)	2(10%)	1 (5%)	1 (5%)
<0.5 fold	0 (0%)	0 (0%)	2 (10%)	7 (33%)	0 (0%)	1 (5%)	0 (0%)
AFE	1.46	1.10	0.83	0.63	1.19	0.86	1.06
**Monkey (*n* = 25)**							
0.5–2 fold	23 (92%)	23 (92%)	23 (92%)	23 (92%)	23 (92%)	23 (92%)	23 (92%)
0.5–1.5 fold	17 (68%)	23 (92%)	23 (92%)	23 (92%)	23 (92%)	23 (92%)	23 (92%)
>2 fold	0 (0%)	0 (0%)	0 (0%)	0 (0%)	0 (%)	0 (%)	0 (%)
<0.5 fold	2 (8%)	2 (8%)	2 (8%)	2 (8%)	2 (8%)	2 (8%)	2 (8%)
AFE	1.03	0.89	0.76	0.66	0.70	0.70	0.70

**LBF:** liver blood flow; AFE: average fold error.

**Table 3:** Observed and predicted human dose by different methods.

Mabs	Human Observed Dose (mg/kg)	Predicted Human Dose (mg/kg)				
		Exponent 1.0	Exponent 0.85	HED (Weight)^0.33^	HED (CL)^0.33^	HED (CL)^0.25^
**Mouse (*n* = 13)**						
CTLA4Ig	1–20	8.4	3	1.9	2.9	5
MNRP1685A	2–40	2.9	1	0.7	1	1.8
Cetuximab	92–920	0.3	0.1	0.1	0.1	0.2
EGF/r3	5.7	4.7	1.7	1.1	1.6	2.8
Bevacizumab	0.1–10	2.8	1	0.6	0.9	1.6
onartuzumab	4 to 30	2.9	1	0.7	1	1.8
Canakinumab	0.3–10	2.9	1	0.7	1	1.8
Anti-CD 40	0.5–8	2.9	1	0.7	1	1.8
RSHZ19	0.025–10	0.3	0.1	0.1	0.1	0.2
Pembrolizumab	1–10	2.9	1	0.7	1	1.8
Infliximab	3–20	2.9	1	0.7	1	1.8
Avelumab	1–20	2.9	1	0.7	1	1.8
Adalimumab	0.25–10	1.5	0.5	0.3	0.5	0.9
**Rat (*n* = 12)**						
CTLA4Ig	1–20	4.3	2.1	1.6	2.1	3
MNRP1685A	2–40	4.2	2.1	1.6	2.1	3
CD4-IgG	0.03–1.0	0.1	0.03	0.02	0.03	0.04
Lenercept	0.014–1.43	2.1	1	0.8	1	1.5
CNTO 5285	0.1–10	4.2	2.1	1.6	2.1	3
EGF/r3	5.7	3.4	1.7	1.2	1.6	2.4
Bevacizumab	0.1–10	4.4	2.1	1.6	2	3
Anti-CD 40	0.5–8	4.3	2.1	1.6	2.1	3
RSHZ19	0.025–10	0.4	0.2	0.2	0.2	0.3
Infliximab	3–20	4.3	2.1	1.6	2.1	3
Adalimumab	0.25–10	0.4	0.2	0.2	0.2	0.3
Dupilumab	1–12	2.1	1	0.8	1	1.5
**Monkey (*n* = 15)**						
CTLA4Ig	1–20	5.6	3.8	3.2	3.8	4.6
MNRP1685A	2–40	9.6	6.5	5.6	6.5	7.9
CD4-IgG	0.03–1.0	0.1	0.1	0.05	0.1	0.1
Lenercept	0.014–1.43	3.2	2.2	1.9	2.2	2.6
CNTO 5285	0.1–10	6.4	4.4	3.7	4.3	5.3
Cetuximab	92–920	4.8	3.3	2.8	3.2	4
Bevacizumab	0.1–10	6.4	4.4	3.7	4.3	5.3
onartuzumab	4–30	6.4	4.4	3.7	4.3	5.3
Canakinumab	0.3–10	3.2	2.2	1.9	2.2	2.6
Anti-CD 40	0.5–8	6.4	4.4	3.7	4.3	5.3
RSHZ19	0.025–10	0.6	0.4	0.4	0.4	0.5
Pembrolizumab	1–10	1.9	1.3	1.1	1.3	1.6
Avelumab	1–20	2.6	1.7	1.5	1.7	2.1
Adalimumab	0.25–10	3.2	2.2	1.9	2.2	2.6
Dupilumab	1–12	0.6	0.4	0.4	0.4	0.5

HED: Human-Equivalent Dose.

## Data Availability

Data are available from the cited references.

## Supplementary Materials

The following are available online at https://www.mdpi.com/article/10.3390/antib10030035/s1, Table S1: Physiological parameters used in the calculation of human physiological factor; Table S2: Estimation of human physiological factor.
