# Supplementary material for: A Single Animal Species-Based Prediction of Human Clearance and First-in-Human Dose of Monoclonal Antibodies: Beyond Monkey
Source: Antibodies (Basel). 2021 Sep 5;10(3):35. doi: 10.3390/antib10030035 (PMC8477747; doi:10.3390/antib10030035)
Supplement: Supplementary file 1 [file antibodies-10-00035-s001.zip › antibodies-1244774-supplementary.pdf]

Table S1: Physiological parameters used in the calculation of human physiological factor

Parameters	Mouse	Rat	Monkey	Humans
Body weight (kg)	0.02	0.25	3.5	70
Liver wt (grams)	1.75	10	150	1500
Kidney wt (grams)	0.32	2	25	310
LBF (mL/min)	1.8	13.8	158*	1600
KBF (mL/min)	1.3	9.2	138	1240
LFR (L/hr)**	0.12	1.28	12.29	121.1

LBF = liver blood flow, KBF = Kidney blood flow, LFR = Lymph flow rate
*Based on monkey liver blood flow of 45 mL/min (from reference 4); physiological parameters from reference 5. **From reference S30

Table S2: Estimation of human physiological factor

Parameters	Mouse	Rat	Monkey	Humans
Liver wt (grams)	857	150	10	1500
Kidney wt (grams)	969	155	12	310
LBF (mL/min)	889	116	10	1600
KBF (mL/min)	954	135	9	1240
LFR (L/hr)	1009	94	10	121.1
Average	936	130	10	

Human factor = Human liver wt/mouse or rat or monkey liver wt and so on

The human clearance was predicted as follows:
Assuming a clearance of a mab in the mouse is 0.5 mL/day then the human clearance = 0.5 x 936 = 468 mL/day.

Estimation of human clearance from Lymph flow rate = 0.5 x 1009 = 505 mL/day

Estimation of human clearance from liver blood flow = 0.5 x 889 = 445 mL/day

References:

S1. Hussein M, Bernson JR, Niesvizky R, Munshi N, Matous J., et al. A phase I multidose study of dacetuzumab (SGN-40; humanized anti-CD40 monoclonal antibody) in patients with multiple myeloma. Hematologica. 2010;95:845-8.

S2. de Vos Sven, Forwro-Torres A, Ansell SM, Kahl B, Cheson BD. A phase II study of dacetuzumab (SGN-40) in patients with relapsed diffuse large B-cell lymphoma (DLBCL) and correlative analyses of patient-specific factors. J Hematol Oncol. 2014;7:1-9.

S3. Davis CB, Hepburn TW, Urbanski JJ, Kwok DC, Hart TK, Herzyk DJ, Demuth SG, Leland M, Rhodes GR. Preclinical pharmacokinetic evaluation of the respiratory syncytial virus-specific reshaped human monoclonal antibody RSHZ19. Drug Metab Dispos 1995;23:1028-36.

S4. Everitt DE, Davis CB, Thompson K, DiCicco R, Ilson B, Demuth SG, Herzyk DJ, Jorkasky DK. The pharmacokinetics, antigenicity, and fusion-inhibition activity of RSHZ19, a humanized monoclonal antibody to respiratory syncytial virus, in healthy volunteers. J Infect Dis 1996;174:463-69.

S5. Richter WF, Gallati H, Schiller CD. Animal pharmacokinetics of the tumor necrosis factor receptor-immunoglobulin fusion protein lenercept and their extrapolation to humans. Drug Metab
Dispos 1999;27:21-5.

S6. Che J, Wang H, Chen Z, Li X, Hou Y, Shan C, Cheng Y. A new approach for pharmacokinetics of single-dose cetuximab in rhesus monkeys by surface plasmon resonance biosensor. J Pharm Biomed Anal 2009;50:183-8.

S7. Luo FR, Yang Z, Dong H, Camuso A, McGlinchey K, Fager K, Flefleh C, Kan D, Inigo I, Castaneda S, Rose WC, Kramer RA, Wild R, Lee FY. Correlation of pharmacokinetics with the antitumor activity of cetuximab in nude mice bearing the GEO human colon carcinoma xenograft. J Clin Oncol. 2000;18:904-14.

S8. Fracasso PM, Burris H III, Arquette MA, et al. A phase 1 escalating single-dose and weekly fixed-dose study of cetuximab: pharmacokinetic and pharmacodynamic rationale for dosing. Clin
Cancer Res. 2007;13:986-93.

S9. Srinivas NR, Weiner RS, Shyu WC, Calore JD, Tritschler D, Tay LK, Lee JS, Greene DS, Barbhaiya RH. A pharmacokinetic study of intravenous CTLA4Ig, a novel immunosuppressive agent, in mice. J Pharm Sci 1996;85:296-8.

S10. Srinivas NR, Weiner RS, Warner G, Shyu WC, Davidson T, Fadrowski CG, Tay LK, Lee JS, Greene DS, Barbhaiya RH. Pharmacokinetics and pharmacodynamics of CTLA4lg (BMS-188667), a novel immunosuppressive agent, in monkeys following multiple doses. J Pharm Sci. 1996;85:1-4.
S11. Srinivas NR, Shyu WC, Weiner RS, Warner G, Comereski C, Tay LK, Greene DS, Barbhaiya RH. Assessment of dose proportionality, absolute bioavailability, and immunogenicity response of CTLA4Ig (BMS-188667), a novel immunosuppressive agent, following subcutaneous and intravenous administration to rats. Pharm Res 1997;14:911-6.
S12. Ma Y, Lin BR, Lin B, Hou S, Qian WZ, Li J, Tan M, Ma J, Li BH, Wang H, Wen AD, Guo YJ. Pharmacokinetics of CTLA4Ig fusion protein in healthy volunteers and patients with rheumatoid arthritis. Acta Pharmacol Sin 2009;30:364-71.
S13. Mordenti J, Chen SA, Moore JA, Ferraiolo BL, Green JD. Interspecies scaling of clearance and volume of distribution data for five therapeutic proteins. Pharm Res. 1991;8:1351-9.
S14. Hodges TL, Kahn JO, Kaplan LD, Groopman JE, Volberding PA, Amman AJ, Arri CJ, Bouvier LM, Mordenti J, Izu AE, et al. Phase 1 study of recombinant human CD4-immunoglobulin G therapy of patients with AIDS and AIDS-related complex. Antimicrob Agents Chemother. 1991;35:2580-6.
S15. Xin Y, Bai S, Damico-Beyer LA, Jin D, Liang WC, Wu Y, Theil FP, Joshi A, Lu Y, Lowe J, Maia M, Brachmann RK, Xiang H. Anti-neuropilin-1 (MNRP1685A): unexpected pharmacokinetic differences across species, from preclinical models to humans. Pharm Res. 2012;29:2512-21.
S16. Chakraborty A, Tannenbaum S, Rordorf C, Lowe PJ, Floch D, Gram H, Roy S. Pharmacokinetic and pharmacodynamic properties of canakinumab, a human anti-interleukin-1β monoclonal antibody. Clin Pharmacokinet. 2012;51:e1-18.
S17. Xiang H, Bender BC, Reyes AE, Merchant M, Jumbe NL, Romero M, Davancaze T, Nijem I, Mai E, Young J, Peterson A, Damico-Beyer LA. Onartuzumab (MetMAb): using nonclinical pharmacokinetic and concentration-effect data to support clinical development. Clin Cancer Res 2013;19:5068-78.
S18. Salgia R, Patel P, Bothos J, Yu W, Eppler S, Hegde P, Bai S, Kaur S, Nijem I, Catenacci DV, Peterson A, Ratain MJ, Polite B, Mehnert JM, Moss RA. Phase I dose-escalation study of onartuzumab as a single agent and in combination with bevacizumab in patients with advanced solid malignancies. Clin Cancer Res. 2014;20:1666-75.
S19. Ducongé J, Ferna´ndez-Sa´nchez E, Alvarez D. Interspecies scaling of the monoclonal anti-EGFreceptor ior EGF/r3 antibody disposition using allometric paradigm: Is it really suitable? Biopharm Drug Dispos 2004;25:177-86.
S20. Nnane IP, Xu Z, Zhou H, Davis HM. Non-Clinical Pharmacokinetics, Prediction of Human Pharmacokinetics and First-in-Human Dose Selection for CNTO 5825, an Anti-Interleukin-13 Monoclonal Antibody. Basic Clin Pharmacol Toxicol 2015;117:219-25.

S21. Lindauer A, Valiathan CR, Mehta K, Sriram V, de Greef R, Elassaiss-Schaap J, de Alwis DP. Translational Pharmacokinetic/Pharmacodynamic Modeling of Tumor Growth Inhibition Supports Dose-Range Selection of the Anti-PD-1 Antibody Pembrolizumab. CPT Pharmacometrics Syst Pharmacol. 2017;6:11-20.
S22. Pembrolizumab: FDA Pharmacology Review, CENTER FOR DRUG EVALUATION AND RESEARCH. BLA 125514. 2014.
S23. FDA package insert. KEYTRUDA (pembrolizumab) for intravenous use, 2014.
S24. Infliximab: FDA Pharmacology Review, CENTER FOR DRUG EVALUATION AND RESEARCH. BLA 761054. 2016.
S25. FDA package insert. REMICADE (infliximab), 1998.
S26. Avelumab. Assessment Report. Committee for Medicinal Products for Human Use (CHMP). European Medicinal Agency (EMA), 2017.
S27. Deng R, Loyet KM, Lien S, Iyer S, DeForge LE, Theil FP, Lowman HB, Fielder PJ, Prabhu S.Pharmacokinetics of humanized monoclonal anti-tumor necrosis factor-{alpha} antibody and its neonatal Fc receptor variants in mice and cynomolgus monkeys. Drug Metab Dispos. 2010;38:600-5.
S28. Park Y, Kim N, Choi J, Park M-H, Lee BI, et al. Qualification and Application of a Liquid Chromatography-Quadrupole Time-of-Flight Mass Spectrometric Method for the Determination of Adalimumab in Rat Plasma. Pharmaceutics 2018;61:2-12.
S29. FDA package insert. HUM IRA (adalimumab) Injection, Solution for Subcutaneous use, 2002.
S30. Dupilumab. Pharmaceutical Evaluation Division, Pharmaceutical Safety and Environmental Health Bureau Ministry of Health, Labour and Welfare, Japan, 2017.
S31. Zhao J, Cao Y, Jusko WJ. Across-Species Scaling of Monoclonal Antibody Pharmacokinetics Using a Minimal PBPK Model. Pharm Res. 2015;32: 3269-81.
